# Preclinical Evaluation of Intraoperative Low-Energy Photon Radiotherapy Using Spherical Applicators in Locally Advanced Prostate Cancer

**DOI:** 10.3389/fonc.2015.00204

**Published:** 2015-09-15

**Authors:** François Buge, Sophie Chiavassa, Chloé Hervé, Jérôme Rigaud, Grégory Delpon, Stéphane Supiot

**Affiliations:** ^1^Department of Urology, Centre Hospitalier Universitaire de Nantes, Nantes, France; ^2^Centre de Recherche en Cancérologie Nantes-Angers, INSERM U892, Université de Nantes, Nantes, France; ^3^Department of Medical Physics, Institut de Cancérologie de l’Ouest, St-Herblain, France; ^4^Department of Radiation Oncology, Institut de Cancérologie de l’Ouest, St-Herblain, France

**Keywords:** prostate cancer, prostatectomy, radiotherapy, intraoperative radiotherapy, combined modality therapy

## Abstract

**Background:**

Surgery plus adjuvant radiotherapy is standard care for locally advanced prostate cancer (stage pT3R1). Intraoperative low-energy photon radiotherapy offers several advantages over external beam radiotherapy, and several systems are now available for its delivery, using spherical applicators, which require only limited shielding. The aim of this study was to evaluate the feasibility of this technique for the prostate bed.

**Materials and methods:**

Applicators were assessed using MRI image data and cadaveric dissection. In cadavers, targeted tissues, defined as a urethral section, both neurovascular bundle sections, the bladder neck and the beds of the seminal vesicles, were marked with metallic surgical clips. Distances between clips and applicator were measured using CT. A dosimetric study of the application of 12 Gy at 5 mm depth was performed using CT images of prostatectomized cadavers.

**Results:**

Using MRI images from 34 prostate cancer patients, we showed that the ideal applicator diameter ranges from 45 to 70 mm. Using applicators of different sizes to encompass the prostate bed in nine cadavers, we showed that the distance between target tissues and applicator was <2 mm for all target tissues except the upper extremity of the seminal vesicles (19 mm). Dosimetric study showed a good dose distribution in all target tissues in contact with the applicator, with a low probability of rectum and bladder complication.

**Conclusion:**

Intraoperative radiotherapy of the prostate bed is feasible, with good coverage of targeted tissues. Clinical study of safety and efficacy is now required.

## Introduction

Increasing numbers of patients are undergoing surgery for high-risk prostate cancer (1). Despite adequate surgery, half of all patients with locally advanced prostate adenocarcinoma (stage pT3) will present in biochemical relapse in the fifth year after operation, suggesting that many patients may not be curable by surgery alone. Three studies have been evaluated the role of a multi-modal approach that combines surgery with adjuvant irradiation in the prevention of relapse after prostatectomy (2–4). All three studies showed reduction in the rate of biochemical relapse, and one showed better metastasis-free survival and overall survival among patients who had received adjuvant irradiation (2). These studies emphasized that the main mode of relapse in prostate cancer is local and that intensifying local treatment reduces the risk of tumor recurrence (5). The relapse site is primarily anastomotic in more than two-thirds of cases, but may also occur at the level of the bladder neck, and occasionally retrovesically (6). This means that the prostate bed boundaries should be defined anteriorly by the posterior wall of the pubic bone, posteriorly by the anterior wall of the rectum, laterally by the levator ani muscles, caudally by the pelvic floor, and cranially by the level of section of the *vas deferens* (7).

Post-operative irradiation is usually carried out between 3 and 6 months after surgery in order to allow a better sphincter recovery. However, this long-time period exposes the patients to the risk of residual tumor growth and metastatic spread, especially for poorly differentiated tumors. To avoid delayed post-operative radiotherapy, perioperative radiotherapy strategies have been developed. Four studies have shown that it is possible to combine prostatectomy with preoperative radiotherapy at the same doses as those used for rectal cancers, without any increase in perioperative toxicity (8). The complication rate appears comparable to that observed among patients irradiated within 6 months of prostatectomy after long-term follow-up (9). Moreover, three studies have demonstrated the feasibility of intraoperative radiation therapy (IORT) using 7–12 MeV electrons during radical prostatectomy (10–12). Single fraction doses ranging from 10 to 22 Gy were administered immediately before or after prostatectomy. pT3 patients also received an additional dose of 45 Gy to the pelvis after surgery. No increase in long-term complications was observed. The rectum was assessed intraoperatively to have received a dose of 3.9 Gy, well below its maximum tolerated dose, which permitted additional external beam radiotherapy (EBRT) if needed (10). However, the use of electrons implies that surgery must be performed in a dedicated shielded operating room or that the patient be moved to a bunker for the treatment delivery.

More recently, the use of low-energy photon IORT has been developed for other cancer types, notably breast cancer (Intrabeam™, Carl Zeiss Meditec, Iena, Germany and Axxent eBx™ System, Xoft, San Jose, CA, USA) (13, 14). Isotropic x-ray irradiation is delivered rapidly to the tissues surrounding the tumor area during surgery. Compared with high-energy photon EBRT, the rapid absorption of low-energy photons limits the dose spread to surrounding tissues, with <35% of the dose delivered to the surface of the applicator at a distance of 10 mm. In contrast to the extensive shielding required for electron therapy, IORT using low-energy photons requires only limited shielding similar to that required for diagnostic x-rays. Moreover, low-energy photons are biologically more destructive than either high-energy photons or electrons, since the relative biological efficacy (RBE) of low-energy photons is estimated between 1.2 and 1.5, whereas the RBE is 1 for high-energy photons or electrons (15). Treatment lasts about 30–50 min, depending on the size of the applicator and the prescribed dose. Intraoperative irradiation is now routinely used in breast cancer patients, with very good clinical short- and long-term efficacy and tolerance (16). This irradiation reduces the delay between surgery and radiotherapy and reduces the travel burden induced by the repeated visits necessary for EBRT.

We performed a preclinical study using both the Intrabeam™ and the Axxent™ systems in prostate cancer patients and in prostatectomized corpses to evaluate the feasibility of intraoperative radiotherapy in prostate cancer.

## Patients and Methods

This study was carried out in accordance with the recommendations of local ethics committee with written informed consent from all subjects.

### Low-energy photon IORT systems

Two systems are commercially available for low-energy photon IORT. Both systems deliver a 50-kV beam.

**Figure 1:**
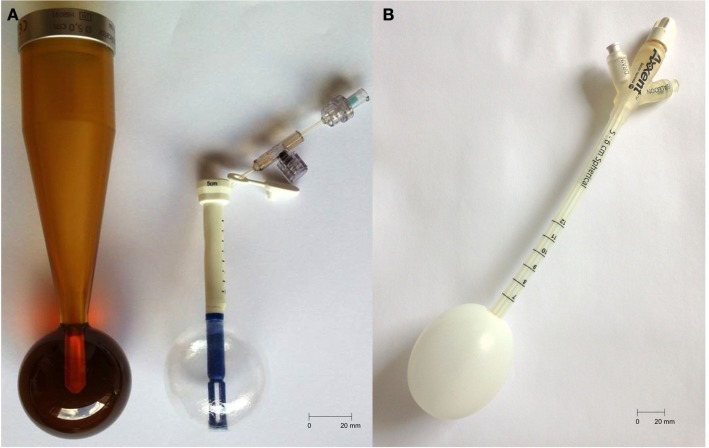
**(A)** Intrabeam: 50 mm rigid (left) and balloon (right) applicators, stalk length = 135 and 65 mm, respectively; **(B)** Xoft: 50–60 mm applicator, stalk length = 250 mm.

Intrabeam™(Zeiss, Germany) uses a miniaturized accelerator introduced in rigid or inflatable spherical applicators, which range from 10 to 50 mm in diameter. The prescribed dose is delivered around the applicator with an isotropic distribution. Axxent™(Xoft, CA, USA) uses a 2.25-mm diameter X-ray source placed in an inflatable spherical or ovoid applicator whose diameter varies from 30 to 70 mm. The source can be moved into the applicator to modulate the dose distribution. This new technology is called “electronic brachytherapy” (eBx). Table 1 and Figure 1 summarize these characteristics.

**Table 1:** **Characteristics of Zeiss Intrabeam™ or Xoft Axxent eBx™ low-energy photon intraoperative radiotherapy (IORT)**.

	Intrabeam™	Axxent eBx™
Photon max energy	50 keV	50 keV
Applicators	Rigid or inflatable: 10–50 mm	Inflatable: 30–70 mm
Stalk length	Rigid: 135 mm	250 mm
	Inflatable: 65 mm	
Dose rate (Gy/min)	0.15	0.6
Delivery time (12 Gy, 5 mm depth, 50 mm applicator) (min)	52.8	21

### Evaluation of the sphericity and dimensions of the prostate bed in prostatectomized cadavers

**Figure 2:**
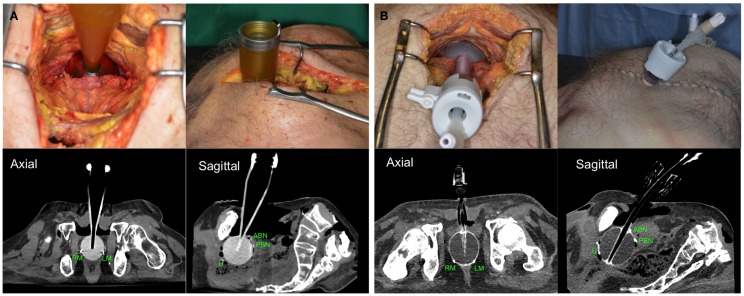
**Positioning of Zeiss Intrabeam™(A) or Xoft Axxent eBx™[(B) through laparoscopic trocar] applicators and CT scans of prostatectomized corpses with radiopaque clips located at different target tissues**. U, urethra; ABN, anterior bladder neck; PBN, posterior bladder neck; LA, left apex; RA, right apex.

Radical prostatectomy without conservation of the neurovascular bundles was performed in nine cadavers in the anatomy laboratory of the University of Nantes. Radio-opaque clips were placed at potential recurrence sites, which were defined as the urethral section, bladder neck, neurovascular bundles, anterior wall of the rectum, and the beds of the seminal vesicles. After prostatectomy and identification of target tissues, applicators (Intrabeam™ or Axxent™ applicators) were inserted in the prostate bed until they were in contact with the urethral section of the pelvic floor. The applicator was then applied to the anterior rectal wall, as closely as possible to the clips marking the neurovascular bundles. The most suitable size was selected visually. Finally, the bladder was lowered to apply the bladder neck against the applicator, and sutured to the pubic symphysis on both sides of the applicator. A CT scan was later performed to measure the distance between the clips and the spherical applicator (Figure 2).

### MRI evaluation of the dimensions of the prostate bed prior to surgery

**Figure 3:**
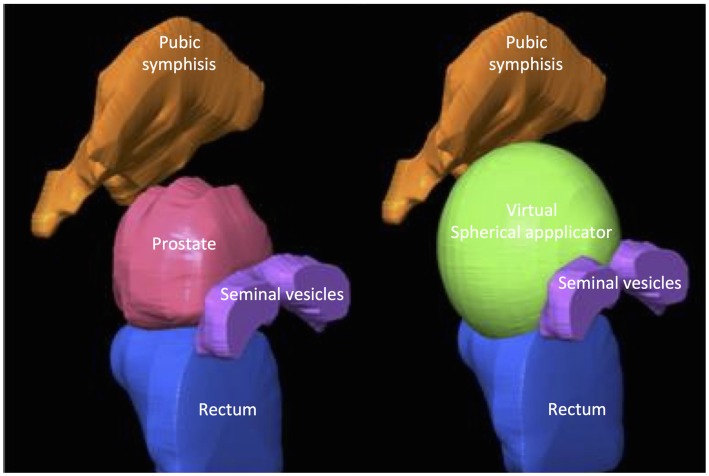
**Graphical representation of the determination of applicator size on MRI images**.

To determine the size of the prostate bed prior to surgery in prostate cancer patients, spheres of increasing diameter (45–70 mm) were generated on 3D-reconstructed T2-weighted sequences (4 mm thick) prostate MRI images using Iplan^®^ (BrainLab, Germany). The pelvic organs – prostate, rectum, seminal vesicles, and the pubic symphysis – were contoured. The smallest sphere to completely encompass the prostate volume was considered the most suitable (Figure 3).

### Dose distribution and estimation of the probability of normal tissue complications

A 50-mm applicator (Intrabeam™ system) and a 50-to 60-mm applicator (Axxent™ system) were inserted in the prostate bed of two cadavers. CT images were then acquired. Pelvic organs at risk (bladder and rectum) were delineated on the images. Dose distribution was calculated using a Monte Carlo simulation for the Intrabeam™ system (17) or using Brachyvision software (Varian medical systems, San Jose, CA, USA) for the Axxent™ system. Dose–volume histograms were computed. A dose of 12 Gy at 5 mm depth, corresponding to 20 Gy at the surface of the applicator, was prescribed, similar to that used for breast cancer IORT (16) and prostate bed IORT using electrons (10, 11). The dose distribution was computed to calculate normal tissue complication probabilities (NTCP) for rectum and bladder using relevant radiobiological parameters (alpha/beta for rectum and bladder 5.4 and 7.5 Gy, respectively) using EBRT and HDR models similar to those outlined by Takam et al. (18).

## Results

### Evaluation of the shape and dimensions of the prostate bed in cadavers

Nine cadavers without prostate cancer (mean age 83, range 78–92) were dissected and prostatectomy without neurovascular preservation performed. Applicators or plastic spheres were then inserted in the prostate bed. Congruence with the anatomical boundaries was evaluated visually when the sphere came into contact with all target tissues. The best-adapted sphere measured 50 mm in four cases, 60 mm in four cases, and 50–60 mm (ovoid applicator) in one case.

Once the positioning had been optimized visually, CT scans were performed to evaluate the congruence of the applicator to the prostate bed and distances between the applicator and radio-opaque clips were measured. In all cases, the congruence of the applicator to the prostate bed was not affected by the shape of the applicator, whether spherical (Intrabeam™) or slightly ovoid (Axxent™) (Table 2).

**Table 2:** **Distance (millimeter) between radio-opaque clips and applicator surface on CT scan in nine prostatectomized cadavers**.

	#1	#2	#3	#4	#5	#6	#6^a^	#7	#8	#9
Age	82	79	92	90	84	80	80	80	78	83
Applicator size (mm)	60	60	60	60	50	50	50	50	50	50–60
Applicator type	rig. S	rig. S	rig. S	rig. S	rig. IB	rig. IB	rig. IB	rig. IB	inf. IB	inf. Ax
Urethra	–	14	–	–	–	6	–	–	–	–
Ant BN	–	–	–	–	–	–	–	–	–	–
Post BN	–	–	–	–	–	–	–	–	–	–
Retrovesical	–	–	–	–	1.3	–	–	–	nd	1.1
Left apex	1.5	15	–	–	–	3	–	–	–	–
Right apex	–	9	–	–	–	1.5	–	–	4	–
Left NVB	–	10	–	–	nd	–	–	–	nd	–
Right NVB	–	–	–	–	–	–	–	–	nd	–
Left base	–	–	–	–	–	–	–	–	–	–
Right base	–	–	–	–	–	–	–	–	–	–
Left distal SV	19	10	nd	nd	nd	nd	nd	nd	nd	nd
Right distal SV	5	2.5	nd	nd	nd	nd	nd	nd	nd	nd
Rectum	Empty	Full	Empty	Empty	Empty	Full	Empty	Empty	Empty	Empty

*^a^Same cadaver after rectal emptying*.

*rig. S, rigid plastic sphere; rig. IB, rigid intrabeam applicator; inf. IB, inflatable intrabeam applicator; inf. Ax, inflatable Axxent applicator; –, 0 mm; BN, bladder neck; NVB, neurovascular bundles; SV, seminal vesicles; nd, not determined*.

The Axxent™ applicator can easily be inserted in a laparoscopic trocar before placement, allowing it to be used whatever the surgical approach chosen (open or laparoscopic, with or without robotic assistance).

Rectal filling increased the distance between the applicator’s surface and the urethral or neurovascular bundle clips by up to 15 mm (cadavers 2 and 6). After removing rectal stool, this distance reduced to <2 mm (cadaver 6). Clips at the distal extremity of the seminal vesicles were always located more than 2 mm from the applicator’s surface in the first two cadavers, so the marking of this target site was abandoned in the next six cadavers studied. In the other cases, the CT scan measured distance between the applicator’s surface and clips ranged between 0 and 6 mm. The size of the applicator did not influence the distance between the clips and applicator’s surface.

### MRI evaluation of the dimensions of the prostate bed prior to surgery

**Figure 4:**
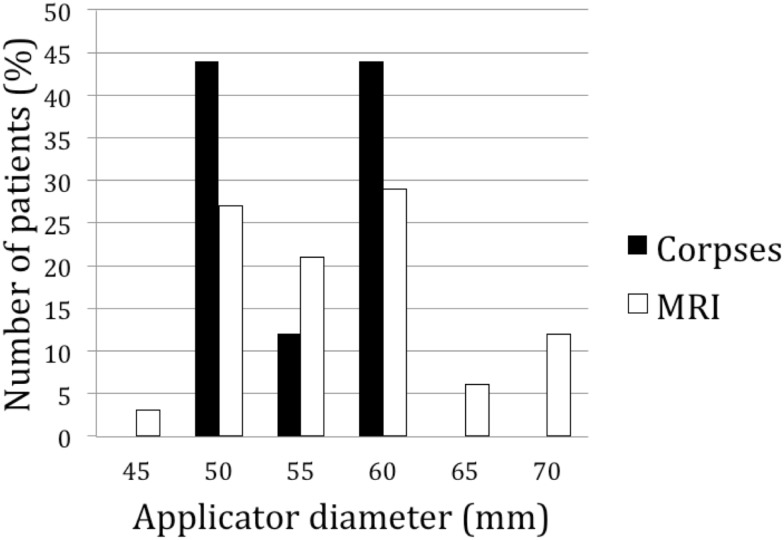
**Distribution of the diameter of the applicator among 9 prostatectomized cadavers (plain bars), and in MRI images from 34 prostate cancer patients (empty bars)**.

To determine the dimensions of the prostate bed in a larger cohort of patients, we simulated the positioning of applicators of different sizes in 34 prostate cancer patients using MRI (Figure 3). Prostate volume ranged from 25 to 106 ml (median = 39.7 ml). After 3D reconstruction and virtual applicator testing, the size ranged between 50 and 70 mm. In 78% of patients, the applicator’s diameter ranged between 50 and 60 mm, confirming the cadaveric measurements (Figure 4). Neither prostatic volumes nor prostate dimensions were predictive of the ideal applicator diameter (data not shown).

### Dose distribution and estimation of the probability of normal tissue complications

**Figure 5:**
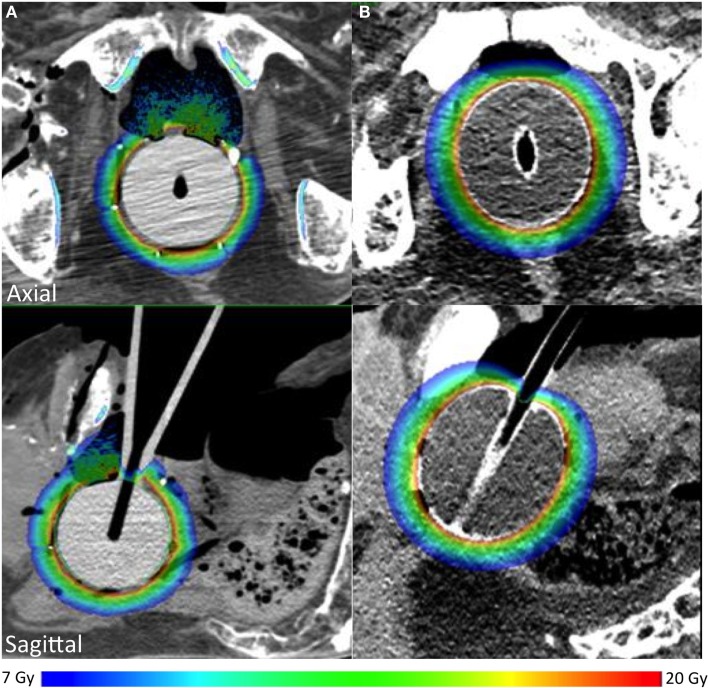
**Dose distribution in targeted tissues using Zeiss Intrabeam™(A) or Xoft Axxent™(B)**.

Dose distribution was calculated in two cadavers in which a 50-mm applicator (Intrabeam™ or Axxent™) had been inserted. A similar dose distribution to target tissues in contact or in the close vicinity of the applicator was obtained (Figure 5). In the cadaver with rectal distension (cadaver 6), the dose to the urethra was decreased by 28% (full rectum: 8.6 Gy; empty rectum: 12 Gy). Using either the EBRT (e) or HDR (h) model, the NTCP for the rectum was 2.3% (e) and 1.0% (h) for the Intrabeam™ irradiation and <1% (e) and (h) for the Axxent™ irradiation. The NTCP for the bladder was 2.8% (e) and 2.0% (h) for the Intrabeam™ irradiation and <1% (e) and (h) for the Axxent™ irradiation. NCTP were lower with (h) because the distances from the applicator to the bladder and the rectum were larger for those cases.

## Discussion

We have shown that low-energy photon IORT using spherical applicators can be adapted for treatment of the prostate bed with the exception of the upper extremity of the seminal vesicles, and that the radiation dose received by the pelvic organs at risk is consistent with a low probability of acute and late toxicity.

The spherical shape of the applicators was suitable for the anatomical configuration of the prostate bed. The applicator shape could be fully spherical (Intrabeam™) or ovoid (Axxent™) without incongruence of the applicator to the prostate bed. However, it is important to empty the rectum since the distance between the applicator’s surface and the urethral section was increased in the cadaver with a full rectum, which significantly reduced the dose to this target tissue.

The applicator positioning was standardized. It was impacted in the rectum in order to be in contact with the neurovascular bundle section. Then, it was applied close to the urethral section. Finally, bladder neck was lowered into contact with the applicator. The choice of the size was visual, testing different applicators. Using this approach (standardized positioning verified visually), CT scan confirmed adequate positioning of the applicator and good dosimetric coverage in all cadavers, with the exception of the one whose rectum was initially full.

Applicators could be rigid (Intrabeam only) or inflatable (Intrabeam and Axxent). This property had no effect neither on positioning in cadaver nor on CT scan image quality. However, inflatable applicators could easily be inserted in a laparoscopic trocar before placement, allowing them to be used whatever the surgical approach chosen (open or laparoscopic, with or without robotic assistance). Among inflatable applicators, we found that the Axxent one seemed more convenient because (1) the longer stalk allows a better adaptation to the anatomy of patients and (2) the four times higher dose rate should reduce operating time.

We selected areas at risk – positive margins and/or areas frequently involved in local recurrences (19) – as target tissues, and determined whether all target tissues would receive the prescribed dose. Our results showed that all target tissues would be irradiated at the same dose, including the proximal, but not the distal, part of the seminal vesicles. Invasion into the seminal vesicles is usually limited to the proximal part; the distal part is invaded only in 20% of pT3b cases (20), so IORT would only miss a very limited number of remaining tumor cells. Moreover, pT3b patients are at high risk of metastatic disease (21), which implies that systemic therapies would be probably more important than increased local treatment in this clinical situation.

We simulated a dose of 12 Gy at a 5-mm depth, 20 Gy at the surface of the applicator. The 5-mm depth encompassed all radio-opaque clips, which suggests that all target tissues would receive a dose ranging between 12 and 20 Gy, which is equivalent to 36–92 and 46–123 Gy in 2 Gy fractions for alpha/beta equal, respectively, to 3 and 1.5 Gy (22). We selected a dose of 12 Gy at a 5-mm depth since (1) this dose is routinely used for breast IORT (16), (2) no increased acute and late toxicity was seen in clinical series following a 12 Gy irradiation using IORT with electrons, and (3) it may be combined with post-operative irradiation without increasing acute and late toxicity (10–12). Higher single-dose treatment seems to be well tolerated, since 22 Gy in one single fraction of IORT using electrons did not increase perioperative or late toxicity (12).

Both Intrabeam and Axxent systems deliver low-energy photons (50 keV) limiting shielding measures necessary to avoid medical staff exposure. In our institution, we performed measures with Intrabeam system (40 mm applicator, 50 kV, 40 μA) before beginning IORT for breast cancer. We found a dose rate of 1700 μSv/h at 1 m from the source and 1.6 μSv/h behind a movable lead shield at 3 m from the source. As dose rate decreases when applicator diameter increases, the exposure should be lower with a 50-mm applicator. Slightly similar dose rates are observed with Axxent system with a dose rate of 2000 μSv/h at 30 cm from the treated area that decreases more than 95% behind a movable lead shield (23).

The main limitation of our study is the determination of the NTCP. NTCP models are based on fractionated irradiation. To determine the complication probabilities of low-energy photon IORT, we assumed that this model could be applied to high single-dose irradiation, which is not definitively proven (24). This model has been developed for high-energy photons; models for low-energy photons are lacking. We could not specifically determine the probability of urethral stenosis using this NTCP model. One adjuvant radiotherapy study showed an increased frequency of urethral stenosis (2), whereas two other studies did not show any differences between adjuvant irradiation and observation (3, 4). A clinical phase I study will be required to carefully evaluate the perioperative toxicity of low-energy photon IORT.

## Conclusion

Our study suggests that low-energy photon IORT, using spherical applicators, is feasible during radical surgery for localized prostate cancer. Selection of the precise applicator and low-energy photon IORT machine will depend on patient characteristics (prostate volume, prostate depth). The current indication for adjuvant radiotherapy is pT3 disease based on the post-operative pathological examination (25). It would be reasonable to select patients who might benefit from low-energy photon IORT prior to surgery based on the probability of pT3 stage disease: according to PSA level, Gleason score >7, or cT3a disease with resectable disease or extra-capsular extension on MRI, though patients with T3b disease should be excluded (26).

## Author Contributions

All the authors equally contributed to the preparation of this manuscript.

## Conflict of Interest Statement

The authors declare that the research was conducted in the absence of any commercial or financial relationships that could be construed as a potential conflict of interest.

## Abbreviations

CT, computed tomography; EBRT, external beam radiotherapy; IORT, intraoperative radiation therapy; NTCP, normal tissue complication probabilities.
